# On the advancement of highly cited research in China: An analysis of the Highly Cited database

**DOI:** 10.1371/journal.pone.0196341

**Published:** 2018-04-24

**Authors:** John Tianci Li

**Affiliations:** Department of Science, The Wheatley School, Old Westbury, NY, United States of America; Universidad de las Palmas de Gran Canaria, SPAIN

## Abstract

This study investigates the progress of highly cited research in China from 2001 to 2016 through the analysis of the Highly Cited database. The Highly Cited database, compiled by Clarivate Analytics, is comprised of the world’s most influential researchers in the 22 Essential Science Indicator fields as catalogued by the Web of Science. The database is considered an international standard for the measurement of national and institutional highly cited research output. Overall, we found a consistent and substantial increase in Highly Cited Researchers from China during the timespan. The Chinese institutions with the most Highly Cited Researchers- the Chinese Academy of Sciences, Tsinghua University, Peking University, Zhejiang University, the University of Science and Technology of China, and BGI Shenzhen- are all top ten universities or primary government research institutions. Further evaluation of separate fields of research and government funding data from the National Natural Science Foundation of China revealed disproportionate growth efficiencies among the separate divisions of the National Natural Science Foundation. The most development occurred in the fields of Chemistry, Materials Sciences, and Engineering, whereas the least development occurred in Economics and Business, Health Sciences, and Life Sciences.

## Introduction

The Highly Cited Researchers database (http://hcr.stateofinnovation.com/) is comprised of the most influential researchers in the 22 Essential Science Indicator fields (ESI) of the Web of Science [1]. Citation analysis measures the propagation of research results, and the Highly Cited Researchers are considered to be the most influential in their field, with respect to the dissemination of their publications. The database is an easy standard to measure the performance of the researchers, along with the performance of their institutional affiliations and nations, and has been commonly used in rankings [2–4]. The Highly Cited database was previously the property of Thomson Reuters, and is now a product of Clarivate Analytics. It is created by cataloging the papers in the top 1% based upon citation count in the corresponding field in the Science Citation Index Expanded, which does not include conference proceedings. Using this list of papers, the authors of the papers are then selected, based upon the number of highly cited papers they have published and the number of citations that their highly cited papers received. In the case that two authors have the same name, the papers are examined, along with the institution and publication history of the author to disambiguate the authors and award proper credit. For the database, whole counting is utilized [5]. Whole counting credits each author, including collaborating authors, for highly cited papers [6]. Although this leaves the possibility that the increase of the number of total Highly Cited Researchers is due to increased collaboration, this is relatively insignificant, because the total number of Highly Cited Researchers in the world is fairly constant across the years, and dividing the Highly Cited Researcher frequency for China by the total Highly Cited Researcher frequency in the world for each field prior to comparison normalizes China’s relative impact on a per field basis, and accounts for both collaboration and field growth.

An increase in the number of Highly Cited Researchers is expected, as the percentage of articles published in journals catalogued by the Web of Science by Chinese researchers has nearly tripled in the 21^st^ century [7]. China is known to be focused on commercial research, but increasing importance of rankings has shifted its perspective on research from an economic standpoint, to the objective of becoming increasingly competent in international rankings based upon research performance. Because of China’s deep roots in the application of the physical sciences, most of the research is concentrated in fields such as Physics, Chemistry, Materials Science, and Engineering [8]. Recently the focus has also expanded to biotechnology, as seen in China’s $9 billion dollar fifteen year precision medicine initiative approved in 2016, and research growth in biology is expected in the near future [9].

China’s rapid economic growth has propelled funding in the sciences, and it has become the second in the world by annual number of publications and amount of research funding. In physical science fields such as Materials Science and Chemistry, China’s publication count has exceeded the publication count of the United States. However, it is still lagging in publications in Biology and Social Sciences [10]. The large volume of publications has led to China’s increasing prominence in highly cited research, as it now produces approximately a fifth of the world’s most highly cited papers. China currently has approximately 1.5 million researchers, which exceeds that of the United States, which only has 1.3 million [11].

The Natural National Science Foundation of China (NSF) is the primary grant-awarding source of basic and applied research funding in China, being responsible for distributing the money that the Central Government dedicates for scientific research to the individual researchers and projects. Other branches of government, such as the Ministry of Science and Technology, are centered around national scale projects, such as directing scientific development, advancing higher education, optimizing energy and resources, and performing large scale military and industrial research to benefit the economy. NSF collaborates with the branches of government to fund research, and allocates the money available as the National Natural Science Fund. The Chinese Academy of Sciences, the primary research institution of China, is mainly supported by the NSF.

China significantly increased research funding and activity in the 21^st^ century. According to the NSF, from 2001 to 2016, China multiplied its general government funding for research by 12.8 fold, and its number of research projects by 3.8 fold [12]. The economic strength of the country also increased in the same time period, measured by an 8.5 fold increase in Gross Domestic Product (GDP) from 2001 to 2016 [13, 14]. Correspondingly, China has seen substantial increases in its prominence in the Highly Cited Researchers database since 1989 [3], and throughout the 21^st^ century [2, 15]. In 2001, China only had seven entries. However, by 2014, there were 160 entries in the Highly Cited database with a primary affiliation located in Mainland China, and Mainland China’s world rank improved to fourth in the world [15]. Mainland China, as in previous studies, does not include Taiwan, Hong Kong, and Macau [15].

This study investigated the growth of highly cited research in China from 2001 to 2016 utilizing the 2001, 2014, 2015, and 2016 Highly Cited Researchers databases, along with government funding data for the divisions of science from the NSF [1, 12]. The NSF grouped the fields of research into seven divisions prior to 2010, whereas in 2010, it separated the preexisting Health Sciences branch from the Life Sciences Division, which led to the current eight divisions. We found momentous overall growth in the initial overall evaluation. Further analysis of development in each of the divisions revealed disproportionate improvements in the separate fields of research, especially when economic factors such as government funding allocation and number of projects were considered.

## Methodology

The 2001, 2014, 2015, and 2016 Highly Cited Researchers lists were downloaded as Excel spreadsheet files from the Clarivate Analytics Highly Cited Researchers website archive (http://www.hcr.stateofinnovation.com/page/archives). In Excel, we restricted the datasets to only researchers with primary affiliations located in Mainland China. Researchers from Taiwan, Hong Kong, and Macau were not included. We tallied the total number of researchers, and ranked the institutions by number of researchers in all fields for each year, thus generating Tables 1, 2, 3 and 4. Table 1 included all institutions with a Highly Cited Researcher, whereas Tables 2, 3 and 4 only included the organizations in Mainland China with more than one Highly Cited Researcher, which restricted the data to the top organizations. For 2016, as shown in Table 4, the number of academic faculty staff was obtained, and the number of Highly Cited Researchers per 1000 academic faculty staff was calculated. The number of academic faculty staff was obtained from the US News World Report, QS TopUniversities, and the websites of the institutions [16–19]. The number of Highly Cited Researchers per 1000 academic faculty staff is likely insignificant. However, the number of academic faculty staff allows for a rough estimate of the size of institutions. Ranking the institutions in each of the 22 ESI fields by Highly Cited Researchers in the specific fields led to Tables 5, 6, 7 and 8.

**Table 1:** 2001 rank of research institutions in China by number of Highly Cited Researchers.

2001 Rank	Institution (No. of researchers)
1	Beijing University of Aeronautics and Astronautics (1)
1(tie)	Chinese Academy of Sciences (1)
1(tie)	Fudan University (1)
1(tie)	Donghua University (1)
1(tie)	Jilin University (1)
1(tie)	Shanghai Jiao Tong University (1)
1(tie)	University of Science and Technology of China (1)

**Table 2:** 2014 rank of research institutions in China by number of Highly Cited Researchers.

2014 Rank	Institution (No. of researchers)
1	Chinese Academy of Sciences (50)
2	University of Science and Technology of China (6)
3	BGI Shenzhen (5)
3(tie)	Peking University (5)
5	Tsinghua University (4)
6	Fudan University (3)
6 (tie)	Harbin Institute of Technology (3)
6 (tie)	Shanghai Jiao Tong University (3)
6 (tie)	Southeast University (3)
6 (tie)	Sun Yat-Sen University (3)
6 (tie)	Xian Jiao Tong University (3)
12	Central South University (2)
12 (tie)	Lanzhou University (2)
12 (tie)	South China University of Technology (2)
12 (tie)	Zhejiang University (2)

**Table 3:** 2015 rank of research institutions in China by number of Highly Cited Researchers.

2015 Rank	Institution (No. of researchers)
1	Chinese Academy of Sciences (29)
2	Peking University (7)
3	Tsinghua University (5)
4	BGI Shenzhen (4)
4(tie)	China University of Geosciences (4)
4(tie)	University of Science and Technology of China (4)
4(tie)	Harbin Institute of Technology (4)
4(tie)	Zhejiang University (4)
9	Fudan University (3)
9(tie)	Northeast Normal University (3)
9(tie)	Soochow University (3)
9(tie)	South China University of Technology (3)
9(tie)	Southeast University (3)
9(tie)	Xian Jiao Tong University (3)
15	Donghua University (2)
15 (tie)	Shanghai Jiao Tong University (2)
15 (tie)	Sun Yat-Sen University (2)
15 (tie)	University of Electronic Science and Technology of China (2)

**Table 4:** 2016 rank of research institutions in China by number of Highly Cited Researchers.

2016 Rank	Institution (No. of researchers)	Number of Academic Faculty Staff	Number Highly Cited Researchers per 1000 Faculty
1	Chinese Academy of Sciences (25)	21200	1.18
2	Tsinghua University (10)	2936	**3.41**
3	Peking University (6)	4672	1.28
3(tie)	Zhejiang University (6)	3005	**2.00**
5	South China University of Technology (5)	2467	**2.03**
5(tie)	University of Electronic Science and Technology of China (5)	2076	**2.41**
7	BGI Shenzhen (4)	3000	1.33
7(tie)	China University of Geosciences (4)	3037	1.32
7(tie)	Fudan University (4)	2690	1.49
7(tie)	Harbin Institute of Technology (4)	2970	1.35
11	Chinese Academy of Agricultural Sciences (3)	4000	0.75
11 (tie)	University of Science and Technology of China (3)	1832	1.64
13	Beijing Normal University (2)	1734	1.15
13 (tie)	Donghua University (2)	1307	1.53
13 (tie)	Harbin Engineering University (2)	3160	0.63
13 (tie)	Liaoning University of Technology (2)	480	**4.17**
13 (tie)	Nanjing University of Aeronaut & Astronaut (2)	1398	1.43
13 (tie)	Northeast Normal University (2)	1555	1.29
13 (tie)	Shanghai Jiao Tong University (2)	2967	0.67
13 (tie)	Soochow University (2)	2868	0.70
13 (tie)	Sun Yat-Sen University (2)	3078	0.65

**Table 5:** 2001 number of Highly Cited Researchers in each ESI field from research institutions in China.

ESI category	Total No. in 2001	Top Institution (No. of researchers)
Engineering	1	Donghua University (1)
Geosciences	1	Chinese Academy of Sciences (1)
Materials Science	4	Beijing University of Aeronautics and Astronautics (1) Fudan University (1) Jilin University (1) University of Science and Technology of China (1)
Physics	1	Shanghai Jiao Tong University (1)

**Table 6:** 2014 number of Highly Cited Researchers in each ESI field from research institutions in China.

ESI category	Total No. in 2014	Top Institution (No. of researchers)
Agricultural Sciences	1	Chinese Academy of Sciences (1)
Biology & Biochemistry	1	Shanghai Jiao Tong University (1)
Chemistry	26	Chinese Academy of Sciences (9) Peking University (4) Fudan University (2) Sun Yat-Sen University (2)
Computer Science	4	Chinese Academy of Sciences (1) Jiangnan University (1) PLA University of Science and Technology (1) Xian Jiao Tong University (1)
Engineering	23	Chinese Academy of Sciences (4) Harbin Institute of Technology (3) Southeast University (3) Central South University (2) Shanghai Jiao Tong University (2)
Environment/Ecology	2	Chinese Academy of Sciences (1) Zhejiang University (1)
Geosciences	8	Chinese Academy of Sciences (3) China University of Geosciences (2) University of Science and Technology of China (2)
Immunology	1	Tsinghua University (1)
Materials Science	25	Chinese Academy of Sciences (14) Fudan University (2) South China University of Technology (2)
Mathematics	11	Lanzhou University (2) China University of Petroleum (1) Hangzhou Normal University (1) Jiaying University (1) Peking University (1) Shanghai Jiao Tong University (1) Shanghai University of Finance and Economics (1) Southeast University (1) Sun Yat-sen University (1) Xiangtan University (1)
Molecular Biology & Genetics	4	BGI Shenzhen (4)
Neuroscience & Behavior	1	Tsinghua University (1)
Pharmacology & Toxicology	3	Chinese Academy of Sciences (3)
Physics	13	Chinese Academy of Sciences (6) University of Science and Technology of China (3)
Plant & Animal Science	2	Chinese Academy of Sciences (2)

**Table 7:** 2015 number of Highly Cited Researchers in each ESI field from research institutions in China.

ESI category	Total No. in 2015	Top Institution (No of researchers)
Agricultural Sciences	1	Chinese Academy of Sciences (1)
Biology & Biochemistry	2	BGI Shenzhen (1) Shanghai Jiao Tong University (1)
Chemistry	33	Chinese Academy of Sciences (7) Peking University (5) Northeast Normal University (3) Tsinghua University (3) Fudan University (2) Sun Yat-Sen University (2)
Computer Science	4	University of Electronic Science and Technology of China (2) Sichuan University (1) Southeast University (1)
Engineering	24	Harbin Institute of Technology (4) China University of Geosciences (2) Southeast University (2) Xi An Jiao Tong University (2) Zhejiang University (2)
Environment/Ecology	1	Zhejiang University (1)
Geosciences	8	China University of Geosciences (2) Chinese Academy of Sciences (2)
Materials Science	30	Chinese Academy of Sciences (14) South China University Technology (3) Fudan University (2) Tsinghua University (2)
Mathematics	6	Donghua University (1) Guizhou University (1) Jiangnan University (1) Lanzhou University (1) Shanghai Jiao Tong University (1) Southeast University (1)
Microbiology	1	Chinese Academy of Agricultural Sciences (1)
Molecular Biology & Genetics	3	BGI Shenzhen (3)
Physics	6	Chinese Academy of Sciences (4) Beijing Normal University (1) Peking University (1)
Plant & Animal Science	1	Chinese Academy of Sciences (1)

**Table 8:** 2016 number of Highly Cited Researchers in each ESI field from research institutions in China.

ESI category	Total No. in 2016	Top Institution (No of researchers)
Agricultural Sciences	1	Zhejiang University (1)
Biology & Biochemistry	1	BGI Shenzhen (1)
Chemistry	36	Chinese Academy of Sciences (9) Peking University (5) Tsinghua University (5) Fudan University (2) Northeast Normal University (2) Sun Yat-Sen University (2)
Computer Science	10	University of Electronic Science and Technology of China (3) BGI Shenzhen (1) Chinese Academy of Sciences (1) Hefei University Technology (1) Sichuan University (1) Southeast University (1) Southwestern University of Finance Economics (1) Tsinghua University (1)
Engineering	25	Harbin Institute of Technology (4) Chinese Academy of Sciences (3) Zhejiang University (3) China University of Geosciences (2) Harbin Engineering University (2) Liaoning University of Technology (2)
Environment/Ecology	2	Chinese Academy of Sciences (1) Zhejiang University (1)
Geosciences	10	Chinese Academy of Sciences (3) China University of Geosciences (2)
Immunology	1	Tsinghua University (1)
Materials Science	42	Chinese Academy of Sciences (18) South China University of Technology (5) Tsinghua University (5) Fudan University (3) Nanjing University of Aeronaut & Astronaut (2) Soochow University (2)
Mathematics	7	Beijing Normal University (1) Chinese Academy of Sciences (1) Guizhou University (1) Lanzhou University (1) Shanghai Jiao Tong University (1) Southeast University (1) University of Electronic Science and Technology of China (1)
Molecular Biology & Genetics	4	BGI Shenzhen (3) WuXi NextCODE (1)
Neuroscience & Behavior	1	Beijing Normal University (1)
Physics	5	Chinese Academy of Sciences (4) Peking University (1)
Plant & Animal Science	2	Chinese Academy of Sciences (1) Shanghai Jiao Tong University (1)

Then, we obtained government funding data for 2001 to 2016, as PDF and web-based files from the NSF website (http://www.nsfc.gov.cn/publish/portal0/tab104/) [12]. We calculated the percentage of general government funding that was allocated to each of the divisions of research, as delineated by the NSF, and also the percentage of total projects in each division for 2001, 2006, 2011, and 2016, producing Table 9. To evaluate efficiency for each of the separate divisions, we separated the 22 ESI fields according to the eight divisions of the NSF. Using the funding data previously obtained, along with calculated percentage of Highly Cited Researcher frequency for a division out of the global Highly Cited Researcher frequency from 2014 to 2016, we calculated an efficiency index for each division of the NSF, which is included in Table 10. As in Bornmann 2015, Highly Cited Researcher frequency is defined to be the number of entries which are in the Highly Cited database [2]. Thus, a single researcher in both Chemistry and Materials Science would count twice. Since the database is large, with over 3,000 entries, when considering the percentage of frequency, the uniform error would theoretically cancel out. To calculate the Highly Cited Researcher frequency from 2014 to 2016, we added the frequencies of 2014, 2015, and 2016. Average general funding percentage from 2001 to 2016 for the divisions was obtained by averaging the funding data from all of the years from 2001 through 2016. The national efficiency index for each NSF division was calculated by dividing the Highly Cited Researcher frequency percentage from China of the fields included in the division from 2014 to 2016 by the percentage of general research funding invested in the division from 2001 to 2016. GDP data was obtained from The World Bank and The International Monetary Fund [13, 14].

**Table 9:** Government general funding data from the National Natural Science Foundation of China (Funding and project number data obtained from http://www.nsfc.gov.cn/publish/portal0/tab104/).

Research Fields (Divisions)	2001		2006		2011		2016	
	No. Proj. (%)	Amt. Fund. (%)	No. Proj. (%)	Amt. Fund. (%)	No. Proj. (%)	Amt. Fund. (%)	No. Proj. (%)	Amt. Fund. (%)
Mathematical and Physical Sciences	590 (13.30)	9633 (12.08)	1095 (10.66)	30307 (11.28)	1431 (9.34)	86000 (9.57)	1551 (9.16)	95045 (9.34)
Chemical Sciences	463 (10.44)	8240.4 (10.33)	1109 (10.80)	28848 (10.74)	1490 (9.72)	89500 (9.96)	1576 (9.31)	101082 (9.93)
Life Sciences*	1557 (35.11)	27567 (34.56)	3863 (37.61)	96347 (35.87)	2449 (15.98)	144290 (16.05)	2700 (15.94)	162990 (16.02)
Earth Sciences	489 (11.03)	10531 (13.20)	1064 (10.36)	35158 (13.09)	1391 (9.07)	96790 (10.77)	1573 (9.29)	108260 (10.64)
Engineering and Materials Science	683 (15.40)	13555 (16.99)	1563 (15.22)	43207 (16.09)	2606 (17.00)	156442 (17.40)	2851 (16.84)	176900 (17.93)
Information Sciences	454 (10.24)	7763 (9.73)	1102 (10.73)	26235 (9.77)	1611 (10.51)	95500 (10.62)	1861 (10.99)	108600 (10.67)
Management Sciences	199 (4.49)	2473 (3.10)	475 (4.62)	8493 (3.16)	688 (4.49)	28919 (3.22)	720 (4.25)	34560 (3.40)
Health Sciences*	-	-	-	-	3663 (23.90)	201500 (22.42)	4102 (24.22)	230090 (22.61)
**Total**	4435	79762	10271	268595	15329	898941	16934	1017527

Correlation Coefficient between percentage of projects and percentage of general funding: r = +0.989 (Very strong). The percentage of projects is also approximately equal to percentage of funding.

*The Health Sciences Division was established as a branch off from the Life Sciences Division in 2010.

No. Proj.: Number of projects.

Amt. Fund: Amount of funding [Monetary unit is 10,000 China Yuan renminbi (CNY)].

%: Percentage of value out of total.

**Table 10:** National efficiency in the National Natural Science Foundation divisions.

Research Fields (Divisions) by NSF in China	Avg. % Amt. Fund (2001–2016)	ESI category (No. of Res. 2014–2016 in China)	No. of Res. 2014–2016 in China	Total Res. 2014–2016 in the world	% from China	Efficiency index for funding in China
**Mathematical and Physical Sciences**	10.72	Mathematics (24) Physics (24)	48	677	7.09	0.661
**Chemical Sciences**	10.38	Chemistry (95)	95	618	15.37	1.481
**Life Sciences (including Health Sciences)**	35.44	Agricultural Sciences (3) Biology & Biochemistry (4) Clinical Medicine (0) Environment/Ecology (5) Immunology (2) Microbiology (1) Molecular Biology & Genetics (11) Neuroscience & Behavior (2) Pharmacology & Toxicology (3) Plant & Animal Science (5) Psychiatry & Psychology (0)	36	5532	0.65	0.018
**Earth Sciences**	12.16	Geosciences (26)	26	456	5.70	0.469
**Engineering and Materials Sciences**	16.80	Engineering (72) Materials Science (97)	169	918	18.41	1.096
**Information Sciences**	10.16	Computer Science (18)	18	351	5.13	0.505
**Management Sciences**	3.18	Economics & Business (0)	0	236	0.00	0.000
**Health Sciences**	22.40*	Clinical Medicine (0) Pharmacology & Toxicology (3) Psychiatry & Psychology (0)	3	1897	0.16	0.007

Correlation Coefficient between efficiency and percentage funding: r = -0.3 (insignificant)

Avg. % Amt. Fund (2001–2016): Average percentage of government general fund from 2001 to 2016. (*With the exception of the Health Sciences Division, for which the value is calculated from 2010 to 2016).

No. of Res. 2014–2016 in China: The total frequency of Highly Cited Researchers from 2014 to 2016 in China.

Total Res. 2014–2016 in the world: The total frequency of Highly Cited Researchers from 2014 to 2016 in the world.

% from China: The percentage of Highly Cited Researcher frequency from 2014 to 2016 of China compared to the world.

Efficiency index for funding in China: The percentage of total Highly Cited Researcher frequency from 2014 to 2016 from China, compared to the world frequency, divided by average percentage of general government funding from 2001 to 2016 for the division.

## Results

From 2001 to 2016, the total number of Highly Cited Researchers with a primary affiliation in Mainland China increased, from only seven in 2001, to 112 in 2014, 110 in 2015, and 134 in 2016. The percentage of Highly Cited Researchers from China, out of the total number of Highly Cited Researchers, was 0.099% in 2001, 3.484% in 2014, 3.519% in 2015, and 4.104% in 2016, which indicated that highly cited research output from China steadily increased annually. China’s GDP was $1.339 Trillion in 2001, $10.48 Trillion in 2014, $11.06 Trillion in 2015, and $11.2 Trillion in 2016 [13, 14].

In 2001, China had seven Highly Cited Researchers, each with a different primary institution. The institutions with Highly Cited Researchers in China were Beijing University of Aeronautics and Astronautics, the Chinese Academy of Sciences, Fudan University, Donghua University, Jilin University, Shanghai Jiao Tong University, and the University of Science and Technology of China, with the last being very closely associated with Chinese Academy of Sciences, the main government research institution of China. (Table 1)

By 2014, China had 15 institutions with more than one Highly Cited Researcher. (The numbers in parentheses following the name of the institution is the number of Highly Cited Researchers from the institution for a given year.) The top five institutions were the Chinese Academy of Sciences (50), the University of Science and Technology of China (6), BGI Shenzhen (5), Peking University (5), and Tsinghua University (4). BGI Shenzhen is a genomics and bioinformatics institute that is the principle bioinformatics center of the Chinese Academy of Sciences. (Table 2)

In 2015, China had 18 institutions with more than one Highly Cited Researcher. The top institutions were the Chinese Academy of Sciences (29), Peking University (7), Tsinghua University (5), BGI Shenzhen (4), China University of Geosciences (4), the University of Science and Technology of China (4), Harbin Institute of Technology (4), and Zhejiang University (4). (Table 3)

In 2016, China had 21 institutions with more than one Highly Cited Researcher. The top institutions were the Chinese Academy of Sciences (25), Tsinghua University (10), Peking University (6), Zhejiang University (6), South China University of Technology (5), and the University of Electronic Science and Technology (5). Here, we also included the number of academic faculty staff for each institution with two or more Highly Cited Researchers. Dividing the number of Highly Cited Researchers by the number of academic faculty staff, we obtained a measure of efficiency for the organizations. However, this measure of efficiency is likely unreliable, because most institutions have less than ten Highly Cited Researchers. Thus, Liaoning University of Technology, a second-tier institution with very few academic faculty staff has the highest efficiency calculated. The top five institutions with the highest number of Highly Cited Researchers per faculty have their corresponding ratios bolded. The number of academic faculty staff is simply a rough metric of the sizes of the institutions. (Table 4)

The top Chinese institutions overall, the Chinese Academy of Sciences, Tsinghua University, Peking University, Zhejiang University, the University of Science and Technology of China, and BGI Shenzhen, are all either top ten universities, or in the case of Chinese Academy of Sciences and BGI Shenzhen, primary government research institutions. (Tables 1–4)

In 2001, China only had Highly Cited Researchers in four ESI fields. However, four out of a total of seven Highly Cited Researchers were in Materials Science, with the remaining three in Geosciences, Engineering, and Physics. (Table 5)

In 2014, China had Highly Cited Researchers in the following 15 ESI fields: Agricultural Sciences (1), Biology & Biochemistry (1), Chemistry (26), Computer Science (4), Engineering (23), Environment/Ecology (2), Geosciences (8), Immunology (1), Materials Science (25), Mathematics (11), Molecular Biology & Genetics (4), Neuroscience & Behavior (1), Pharmacology & Toxicology (3), Physics (13), and Plant & Animal Science (2). Looking at the fields which China previously had Highly Cited Researchers in, Materials Science grew from four in 2001 to 25 in 2014, Engineering from one to 23, Physics from one to 13, and Geosciences from one to eight. (Table 6)

In 2015, China had Highly Cited Researchers in the following 13 fields: Agricultural Sciences (1), Biology & Biochemistry (2), Chemistry (33), Computer Science (4), Engineering (24), Environment/Ecology (1), Geosciences (8), Materials Science (30), Mathematics (6), Microbiology (1), Molecular Biology & Genetics (3), Physics (6), and Plant & Animal Science (1). From 2014 to 2015, China lost its Highly Cited Researchers in Pharmacology & Toxicology, Neuroscience & Behavior, and Immunology, which had three, one, and one respectively. It however gained a Highly Cited Researcher in Microbiology, in which it previously had none. The number of Highly Cited Researchers in Chemistry, Materials Science, and Engineering continued to increase. Geosciences remained at eight. The number of Highly Cited Researchers in Physics and Mathematics both decreased to six, from 11 and 13 respectively. (Table 7)

In 2016, China had Highly Cited Researchers in the following 14 fields: Agricultural Sciences (1), Biology & Biochemistry (1), Chemistry (36), Computer Science (10), Engineering (25), Environment/Ecology (2), Geosciences (10), Immunology (1), Materials Science (42), Mathematics (7), Molecular Biology & Genetics (4), Neuroscience & Behavior (1), Physics (5), and Plant & Animal Science (2). From 2015 to 2016, China regained its Highly Cited Researchers in Neuroscience & Behavior, and Immunology, with one Highly Cited Researcher in each. However, it lost its only Highly Cited Researcher in Microbiology. Again, the fields of Chemistry and Materials Science grew as rapidly as in the timespan from 2014 to 2015. Computer Science increased significantly, from four to ten. However, Engineering only increased by one, Geosciences by two, and Mathematics by one. Physics decreased by one, and not much change was observed in other fields. (Table 8)

**Fig 1:**
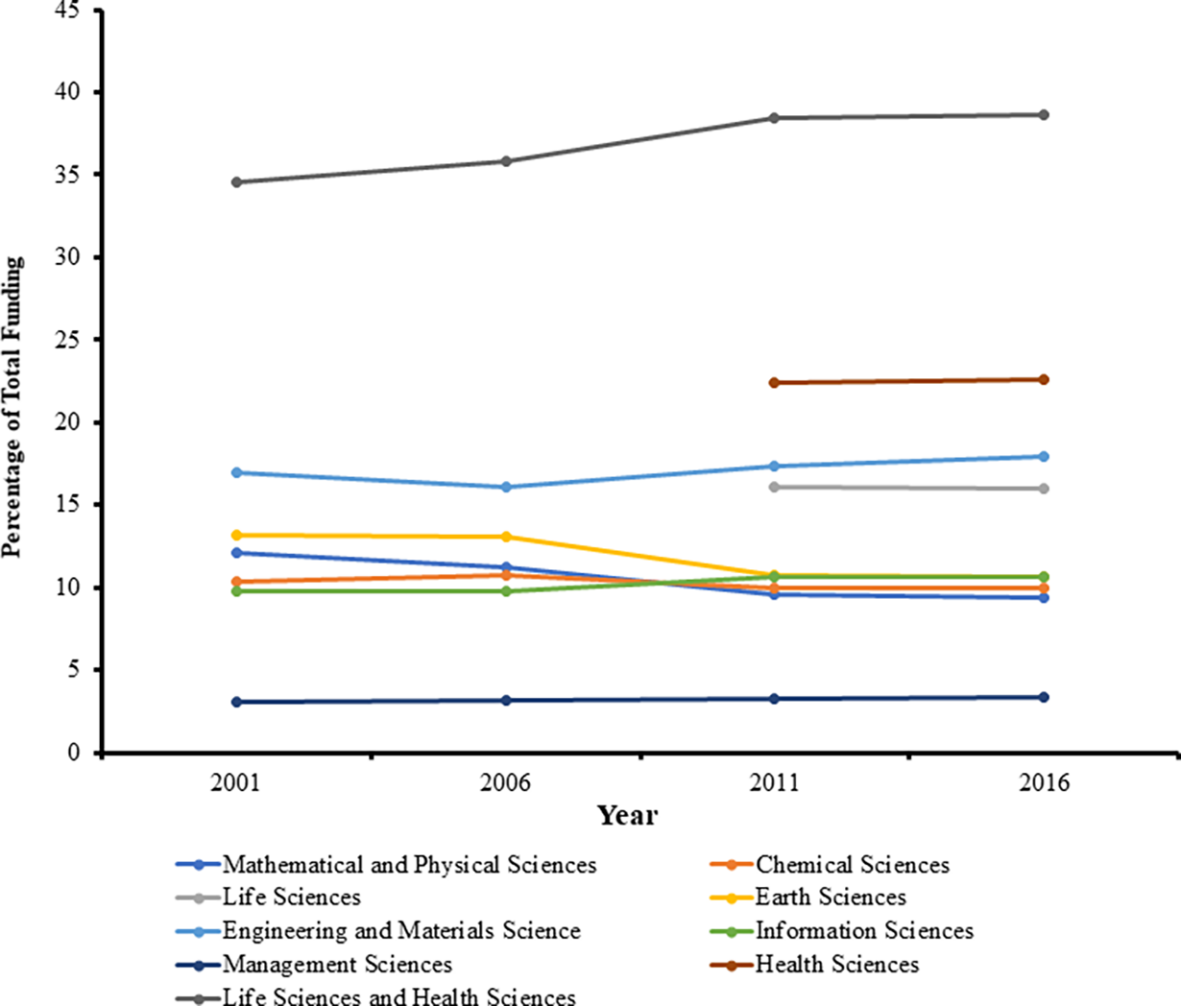
Percentage of total funding allocated to each of the eight NSF divisions. Note that the Health Sciences Division did not exist prior to 2010. Thus, after 2010, the percentage of funding allocated to the Life Sciences Division and the Health Sciences Division combined is approximately equal to the amount allocated to the Life Sciences Division prior to 2010.

Government general funding data from the NSF provided two significant results. From 2001 to 2016, a 15-year period, China multiplied general government funding for research by 12.8 fold, and total number of research projects by 3.8 fold. However, as seen in Fig 1, the amount of funding allocated to each division was disproportionate, with over a third of general government funding being invested into the Life Sciences Division prior to 2010, and the Life Sciences Division and the Health Sciences Division combined after 2010. The Health Sciences Division was a branch of the Life Sciences Division prior to 2010, thus data prior to 2011 for the Health Sciences Division was not available. (Table 9)

**Fig 2:**
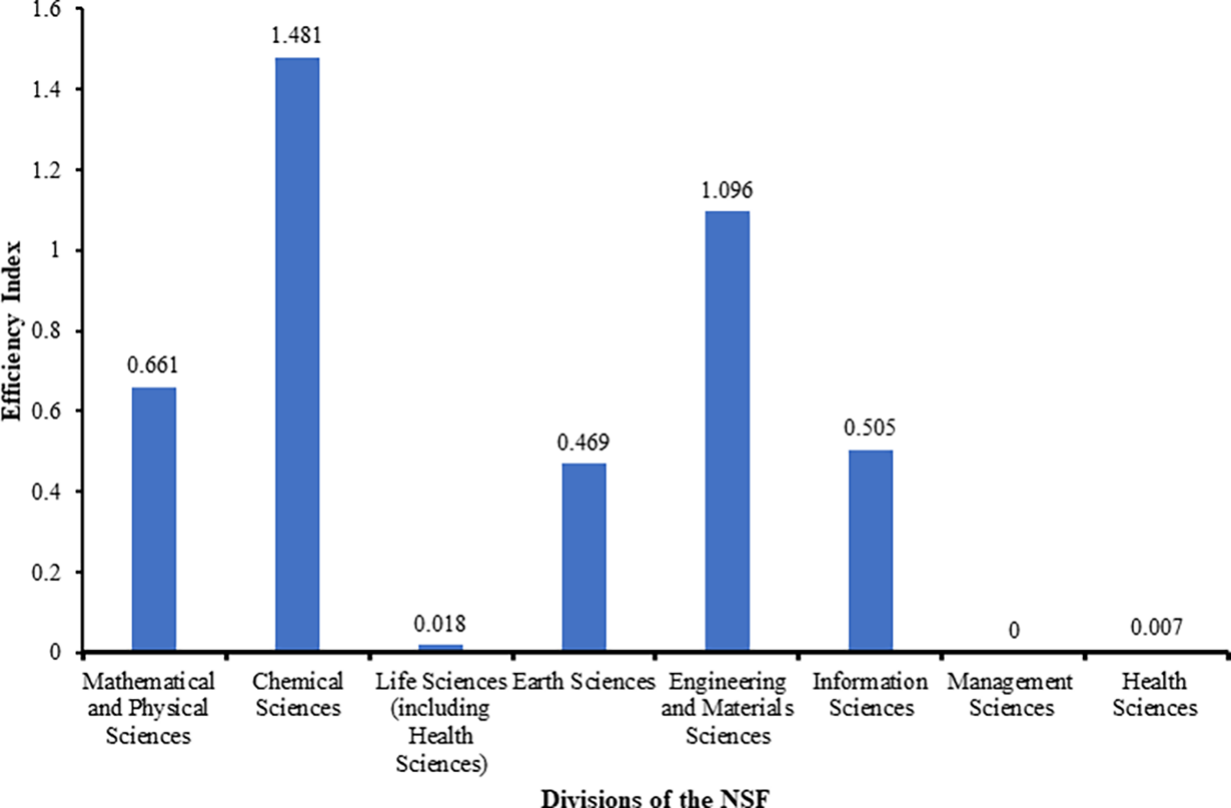
National efficiency indices for the National Natural Science Foundation divisions.

The national efficiency index, calculated as described in the methodology, revealed that the Chemical Sciences Division (1.481) and the Engineering and Materials Science Division (1.096) had relatively high efficiencies when compared to the Management Sciences Division (0), the Life Sciences Division (0.018) and the Health Sciences Division (0.007). The national efficiency indexes are displayed in Fig 2. The Chemical Sciences Division was 82 times more efficient than the Life Sciences Division, and 212 times more efficient than the Health Sciences Division. Similarly, the Engineering and Materials Sciences Division was 61 times more efficient than the Life Sciences Division, and 157 times more efficient than the Health Sciences Division. (Table 10)

## Discussion

China significantly increased highly cited research activity during the timespan from 2001 to 2016. Compared to 2001, by 2016 China had increased the number of active research projects by 3.8 fold, multiplied its government funding by 12.8 fold, and raised its GDP by 8.5 fold, which signified economic growth along with increased interest in research [12–14]. Taking the percentage of Highly Cited Researchers from China out of the total number of Highly Cited Researchers in the world, and comparing it to China’s GDP for the four years, it is seen that highly cited research increased by nearly 4.5 fold for a corresponding two fold increase in GDP. As reflected by the Highly Cited database, China has seen the greatest improvement in highly cited research prominence on the global scale from 2001 to 2016.

A peculiar observation is the yearly decrease of the number of Highly Cited Researchers from the Chinese Academy of Sciences. This phenomenon is primarily driven by university rankings. Numerous rankings of universities, such as the Academic Ranking of World Universities Shanghai Ranking and the US News World Report, use the Highly Cited database to evaluate the performance of an institution [16, 20]. With the recent increase of frequency in updating the Highly Cited Researcher list, and the increased pressure for universities to climb up the rankings, universities are beginning to pay researchers large sums of money to be listed as the primary institution. Notable examples of universities that do this are the King Saud University and the King Abdulaziz University in Saudi Arabia, both of which are known to pay salaries of upwards of three hundred thousand USD for Highly Cited Researchers to list them as their primary institution, or three dollars per citation to be listed as a secondary affiliation [21]. Thus, although many of the researchers are primarily affiliated with the Chinese Academy of Sciences, it is seen that some of them credit another institution as their primary affiliation. Therefore, the number of Highly Cited Researchers that list the Chinese Academy of Sciences as their primary affiliation has decreased, leading to the decline of the Chinese Academy of Sciences in rankings.

China has seen the majority of its growth in the Chemical Sciences Division, and the Engineering and Materials Science Division, which corresponds to the ESI fields of Chemistry, Engineering, and Materials Science. These three fields consistently increased in Highly Cited Researchers, and constituted about two thirds of the Highly Cited Researcher frequency from institutions in China. In 2001, five out of the seven Highly Cited Researchers from China were in Materials Science (4) and Engineering (1) combined. In 2014, 2015, and 2016, the percentage of Highly Cited Researcher frequency for institutions in China that were in the three aforementioned fields are 59%, 72%, and 70% respectively. China has historically been an industrial giant, with much of the country’s economy being reliant upon manufacturing, export-based production, and chemical synthesis and processing businesses. Upon investigation of the economic situation in China, 40.7% of China’s GDP originated from industry, according to the CIA World Factbook [22]. Thus, it is expected that much research and government funding has gone to benefit the economy, in accordance with the Ricardo principle. (Tables 1–8)

From the government general funding data obtained from the NSF, we found consistent increases in the number of active research projects and the amount of general funding provided to each of the divisions. We observed that indeed, a significant portion, approximately 27% of general research funding, went toward the Chemical Sciences Division (10.38%) and the Engineering and Materials Science Division (16.8%). However, we also found that there were two other divisions that were also allocated more than 15% of the government general fund, the Health Sciences Division and the Life Sciences Division, which collectively accounted for 38% of the funds when summed. Prior to 2010, only the Life Sciences Division existed. The Life Sciences Division was allocated approximately 36% of the funding prior to 2010, the year that the Health Sciences branch was established as a separate division. Post 2010, the Life Sciences Division allocation was reduced to 16%, and the Health Sciences Division funding was set to 22.4%, thus summing up to about 38% of the annual total general fund. (Table 9)

For China, the number of projects is directly proportional to the amount of spending. The correlation coefficient between the percentage of funding for a division out of the total amount of funding, and the percentage of projects in a division out of the total number of projects is 0.989, with the two percentages being almost equal. Although spending does not have a direct relation to number of total Highly Cited Researchers, it does have a direct relation to the number of projects. This close relation between percentage of total funding and percentage of total projects results from the funding policy of the NSF. For most types of projects, the amount of grant money provided by the NSF begins at a predetermined fixed amount. Publications are expected to be produced with no further funding, unless if the NSF deems the research to be a national priority. Thus, more funding allows for proportionally more projects, which should correspond to more highly cited publications, and more highly cited publications generally lead to more Highly Cited Researchers [23]. Because the percentage of projects and percentage of funding are approximately equal, the following discussion on efficiency by amount of funding also applies likewise to efficiency by project number.

In the national efficiency by funding evaluation, we found that the most efficient division was the Chemical Sciences Division (1.481), followed by the Engineering and Materials Science Division (1.096). The two divisions were significantly more efficient than the Life Sciences Division (0.018), the Health Sciences Division (0.007), and the Management Sciences Division (0). We found that efficiency corresponds to productivity, as the most efficient and most productive divisions are the same, and the least efficient and least productive divisions are the same. Efficiency was calculated to be inversely correlated to percentage of funding and project number, but only weakly (r = -0.3), indicating that division size does not affect efficiency significantly. (Table 10)

The Mathematical and Physical Sciences Division, Life Sciences Division, Earth Sciences Division, and Information Sciences Division all saw some degree of growth, but not nearly as much as the Chemical Sciences Division and the Engineering and Materials Science Division. The three divisions with the lowest efficiency were found to be the Management Sciences Division (0), Health Sciences Division (0.018), and Life Sciences Division (0.007). We evaluated the Life Sciences Division with the Health Sciences Division included, as the two are closely related, and commonly overlap in operation. Although they were formally separated, the two divisions still function as if they were integrated.

The Management Sciences Division had the lowest efficiency, being zero, as China has not had a single Highly Cited Researcher in the single ESI field included in the division, Economics & Business. However, if it had a single Highly Cited Researcher, the division would fare far better than the next lowest contender, the Health Sciences Division, as the Health Sciences Division received a large portion of funding (22.4%) but only had three Highly Cited Researchers, with all three in the field of Pharmacology & Toxicology, from Chinese Academy of Sciences. China had zero Highly Cited Researchers in Clinical Medicine and Psychiatry & Psychology in 2001, 2014, 2015, and 2016. (Table 10)

China, in the past 16 years, has invested more in the Life Sciences Division (16%) and the Health Sciences Division (22.4%) combined than in any other division. The percentage of government funding invested into the two divisions (38%) is more than double the percentage allocated to the Engineering and Materials Science Division (16.8%), the division with the next highest funding percentage, and with the most Highly Cited Researchers. The efficiency of the Life Science Division (0.018), especially the Health Sciences Division (0.007) branch off, was far too low to be practical, and the amount of money and projects involved in the effort to advance high volume and highly cited research was disproportionate to the results. Very few organizations are productive at outputting highly cited research in the two divisions. For example, BGI Shenzhen is consistently responsible for the large majority of China’s Highly Cited Researchers in the fields of Molecular Biology & Genetics and Biology & Biochemistry. Also, all of the Highly Cited Researchers had a primary institution that is either a top university in China, a private company, or a government research facility which is closely affiliated with Chinese Academy of Sciences. (Tables 1–8 and 10)

Further investigation into the situation reveals that China is attempting to encourage research output in the Life Sciences and Health Sciences Divisions through increased investment in the preexisting medical department based research system, and by promoting a new translational medical research system. In the current situation, it would benefit the country to separate the funding for researchers and physicians, as they are not involved in the same field, nor are they equivalent. Full time physicians in most hospitals in China do not have the time nor the resources to conduct high quality research. Previous efforts, such as those described in Wang et al. (2011) and Leng (2012) [24, 25], have resulted in excessive awarding of grants to full-time physicians, along with systematic misconduct due to the lack of proper management structure [26]. Not only did this lead to inefficient and inappropriate usage of funding, it also put unnecessary pressure on full time physicians, as well as hospital departments, which resulted in incidents of corruption and academic dishonesty. The restructuring of medical research in China has failed, yet the broken system remains unfixed due to the scale of the issue at hand. Thus, China should focus on improving the performance and efficiency of the Life Sciences Division and the Health Sciences Division by reallocating the funds in order to push for high quality instead of high quantity research, and also by learning from the success of the Chemical Sciences Division and the Engineering and Materials Science Division. (Table 10)

The primary purpose of this study is to identify which fields China is and isn’t performing well in relative to the amount of expenditure, and reveals the fields China should invest more heavily into in order to obtain the greatest impact, along with the fields that should undergo organizational and institutional restructuring to optimize their research efficiency and maximize results. Although the efficiency metric serves as a general guideline of research efficiency in terms of impact per unit of expenditure, and should be ideally maximized on a per field basis, the value of the index should not be compared exactly across fields, given that there are limitations, as citations are not the only indicator of research performance. In future studies, other metrics, especially patent application data, should be included and considered as a factor in evaluating scientific advancement. Also, although the Highly Cited database is the largest, most stable, and most comprehensive, there are other smaller rankings and metrics, such as the Google Scholar ranking and the Nature Index. The usage of multiple metrics to evaluate research performance of individual fields of nations allows for more comprehensive results. However, it is very difficult to incorporate data from them for comparison purposes in this study, due to differences in methodology and scope. Doing so will make the paper unnecessarily long for its purpose. Therefore, analyzing and comparing the different databases will be best addressed in a future continuation study.

## Conclusion

Overall, China’s performance in the 21^st^ century is excellent, especially in the ESI fields of Chemistry, Materials Science, and Engineering. During the span of the previous 15 years, China became a major contributor to the global pool of highly cited research. In 2001, China had seven Highly Cited Researchers. By 2014, China became, and has remained as of 2017, one of the top five contributors to the Highly Cited database in the world. Through strategic increases in expenditure, China produced numerous prolific research institutions. Although the growth is disproportionate in the different fields of research, the development is still significant, and demonstrates the high quality highly cited research output capability of the world’s second most economically powerful nation as measured by GDP [13]. However, it will highly benefit the nation to improve efficiency, organizational and operational structure, and project management style by learning from the Chemical Sciences Division and the Engineering and Materials Science Division, especially regarding low efficiency divisions such as the Life Sciences Division and the Health Sciences Division.
